# Malignant syndrome in Parkinson's disease without dopaminergic drug withdrawal

**DOI:** 10.4103/0972-2327.44562

**Published:** 2008

**Authors:** C. J. Suresh Chandran

**Affiliations:** Department of Neurology, Kerala Institute of Medical Sciences, Trivandrum, Kerala, India

**Keywords:** Hyponatremia, malignant syndrome, Parkinson's disease

## Abstract

Malignant syndrome is a rare complication occurring during the course of drug treatment for Parkinson's disease. It resembles neuroleptic malignant syndrome and is characterized by fever, marked rigidity, altered consciousness, leucocytosis and elevated creatine kinase. Malignant syndrome is a potentially fatal condition and awareness of this condition is imperative for prevention and treatment. The commonest precipitating factor is dopaminergic drug withdrawal or dose reduction. We report malignant syndrome (precipitated by hyponatremia) in a case of Parkinson's disease, in the absence of dopaminergic drug withdrawal. A 60-year-old man presented with fever, severe rigidity and altered sensorium following repeated vomiting. On investigation, he was found to have hyponatremia precipitated malignant syndrome. Treatment with hydration, cooling, correction of hyponatremia and dopaminergic drugs reversed his condition. The triad of fever, severe rigidity and altered sensorium should prompt evaluation for malignant syndrome in Parkinson's disease.

## Introduction

Toru *et al*. first described malignant syndrome occurring during the course of the treatment of Parkinson's disease.[1] In Parkinson's disease, malignant syndrome can be precipitated by dopaminergic drug withdrawal, dose reduction, inadequate drug intake, sodium abnormalities, intercurrent infections, dehydration, menstruation etc.[2–5] Hyponatremia precipitated malignant syndrome is being reported in a case of idiopathic Parkinson's disease.

## Case Report

A 60-year-old gentleman with a three-year history of Parkinson's disease was admitted with severe rigidity, fever and altered sensorium following repeated bouts of vomiting. There was no associated abdominal pain, headache or diarrhea. He was on thrice-daily schedule of one tablet of L-Dopa+carbidopa (100 mg + 25 mg), since the last eight months. Prior to the initiation of dopa, he was on amantadine 100 mg twice daily. The Parkinsonian symptoms were reasonably controlled on dopa. He had good compliance and was taking dopa regularly.

**Figure 1:**
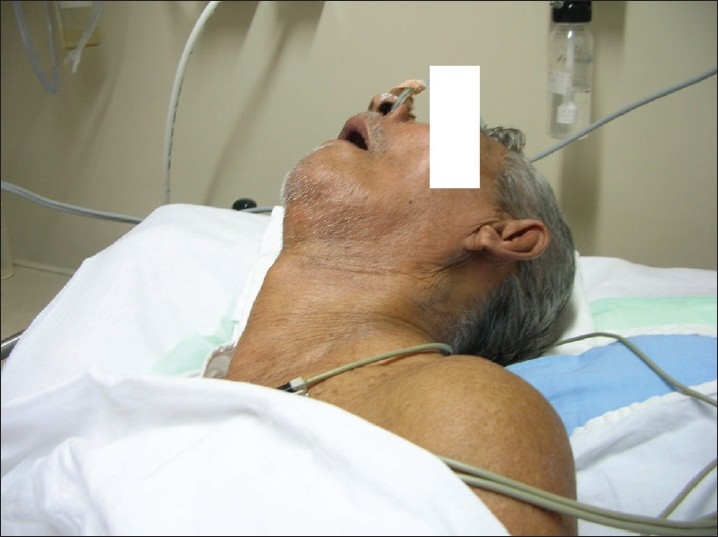
Hyponatremia precipitated malignant syndrome. Note the retrocollis.

**Figure 2:**
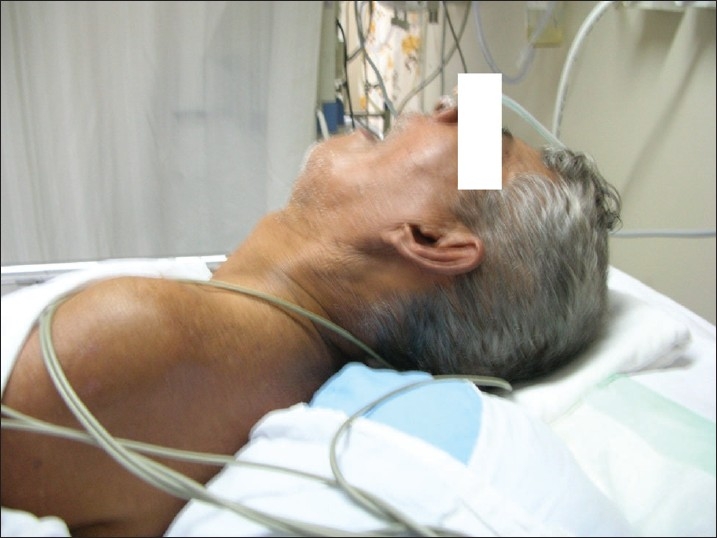
Severe retrocollis in the patient with malignant syndrome

On admission, the patient had a temperature of 101.5°F and was dehydrated and stuporous. He was akinetic rigid, with severe axial and appendicular rigidity and had pronounced and disproportionate retrocollis [Figures 1, 2]. Serum sodium was 109 meq/L. There was leucocytosis (15,000 /mm^3^) and the creatine phosphokinase (CPK) level was elevated (1500 U/L). Non-contrast CT brain was normal, as were the Cerebrospinal fluid (CSF) study, thyroid function test and the rest of the metabolic parameters. No obvious focus of sepsis was detected. Serum osmolality was 265 mOsm/Kg and urine spot sodium concentration was 10 meq/L.

**Figure 3:**
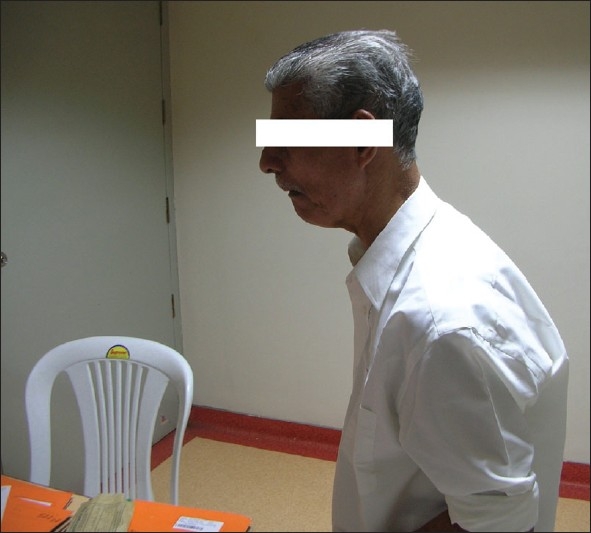
Patient after recovery from malignant syndrome. Note that retrocollis has reversed.

A diagnosis of hypovolemic hyponatremia and malignant syndrome precipitated by hyponatremia was made. Adequate hydration using intravenous fluids and external cooling by tepid sponging was done. Serum sodium was slowly corrected at a rate of 8 meq/L per 24 hours, initially with hypertonic saline (3%) and subsequently with isotonic saline (0.9%). Bromocriptine 2.5 mg thrice daily was added. Dopa was continued in thrice daily schedule. Serum sodium was corrected to 124 meq/L, in forty eight hours. Gradually, sensorium normalized. By the fifth day, serum sodium level normalised and subsequently the patient was mobilized. Over a period of two weeks, retrocollis reverted completely and rigidity decreased. In two weeks, he reverted to the pre morbid state [Figure 3].

## Discussion

The occurrence of malignant syndrome during the course of drug treatment of Parkinson's disease is rare. The abrupt reduction or withdrawal of antiparkinsonian drugs is the most frequent cause, followed by intercurrent infections.[5–7] Other triggering factors include sodium abnormalities, menstruation, inadequate doses, hot weather, dehydration, motor fluctuations (wearing off), antipsychotic drugs, postoperative state and paralytic ileus[2–5] [Table 1]. The exact pathogenesis is not known. Impaired nigrostriatal, hypothalamic and mesolimbic dopaminergic functions could be involved in the pathogenesis.[8] Extremely suppressed central dopaminergic activity occurs in malignant syndrome, as evidenced by low cerebrospinal fluid homovanillic acid levels. This may indicate a narrow safety margin for medication withdrawal in patients with Parkinson's disease. Measuring the CSF levels of monoamine metabolites may provide a means for identifying malignant syndrome (NMS) susceptibility in PD patients.[9,10]

The clinical features are essentially similar to those of neuroleptic malignant syndrome and include severe rigidity, hyperpyrexia, altered consciousness, and dysautonomia. The rigidity in malignant syndrome is usually axial predominant and is present without the maneuver of muscle stretch. On the other hand, Parkinsonian rigidity in the absence of malignant syndrome is induced by muscle stretch. Autonomic dysfunction includes diaphoresis, loss of sweating, tachycardia, fluctuating blood pressure and obstructive ileus. Laboratory evaluation shows leucocytosis and markedly elevated creatine kinase. In advanced cases, complications like rhabdomyolysis, acute renal failure, aspiration pneumonia, pulmonary edema, disseminated intravascular coagulation and deep venous thrombosis are seen.[5,6] The treatment consists of intravenous hydration, external cooling and medications. Levodopa, bromocriptine and dantrolene sodium are found to be effective.[5,7] Bromocriptine 2.5-10 mg thrice daily is the most recommended treatment. Levodopa or dopamine agonists should be continued in the same amount as was taken before the onset of malignant syndrome. If oral or nasogastric feeding is contraindicated due to problems like ileus, intravenous preparations should be used. Steroid pulse therapy for three days (Injection methyl prednisolone) also helps in reducing the illness duration and improving the symptoms.[11] Potential use of NMDA receptor antagonists is being investigated. The management also involves prevention, early recognition and treatment of complications like aspiration pneumonia, deep venous thrombosis (DVT) and renal failure.[5,7] With early treatment, prognosis is usually good. Two thirds of patients recover to go back to the pre malignant syndrome state. Older age, higher Hoehn and Yahr stage during the symptomatic phase of Malignant syndrome (MS), higher akinesia score, and the absence of wearing off phenomenon prior to developing MS are associated with poor outcome.[6]

**Table 1:** Events triggering malignant syndrome in Parkinson's disease

Discontinuation of antiparkinsonian drugs
Abrupt reduction of antiparkinsonian drugs
Poor drug compliance
Intercurrent infections
Hot weather
Dehydration
Sodium abnormalities
Menstruation
Motor fluctuations
Postoperative states
Drugs like antipsychotics
Ileus

In the case of our patient, hyponatremia was the precipitating cause for malignant syndrome and malignant syndrome occurred in the absence of dopamine withdrawal or dose reduction. Our patient had severe retrocollis. Retrocollis is not a usual feature of idiopathic Parkinson's disease and is more typical of Parkinsonism plus syndrome. Such atypical features in Parkinson's disease may be predictors of malignant syndrome. Hyponatremia causes an acute drop in the serum osmolality as a result of which neuronal cell swelling occurs. This might have caused impaired nigrostriatal, hypothalamic and mesolimbic dopaminergic functions, resulting in malignant syndrome.

## Conclusion

Hyponatremia precipitates malignant syndrome in Parkinson's disease. Fever and atypical clinical features in Parkinson's should prompt evaluation for malignant syndrome.

## References

[1] Toru M, Matsuda O, Makiguchu K, Sugano K (1981). Neuroleptic malignant syndrome like state following a withdrawal of antiparkinsonian drugs. J Nerv Ment Dis.

[2] Sechi G, Manca S, Deiana GA, Corda DG, Pisu A, Rosati G (1996). Acute hyponatremia and neuroleptic malignant syndrome in Parkinson's disease. Prog Neuropsychopharmacol Biol Psychiatry.

[3] Stotz M, Thümmler D, Schürch M, Renggli JC, Urwyler A, Pargger H (2004). Fulminant neuroleptic malignant syndrome after perioperative withdrawal of antiparkinsonian medication. Br J Anaesth.

[4] Gordon PH, Frucht SJ (2001). Neuroleptic malignant syndrome in advanced Parkinson's disease. Mov Disord.

[5] Mizuno Y, Takubo H, Mizuta E, Kuno S (2003). Malignant syndrome in Parkinson's disease: Concept and reviews of the literature. Parkinsonism Relat Disord.

[6] Takubo H, Harada T, Hashimoto T, Inaba Y, Kanazawa I, Kuno S (2003). A collaborative study on the malignant syndrome in Parkinson's disease and related disorders. Parkinsonism Relat Disord.

[7] Ikebe S, Harada T, Hashimoto T, Kanazawa I, Kuno S, Mizuno Y (2003). Prevention and treatment of malignant syndrome in Parkinson's disease: A consensus statement of the malignant syndrome research group. Parkinsonism Relat Disord.

[8] Figa Talamanca L, Gualandi C, Di Meo L, Di Battista G, Neri G, Lo Russo F (1985). Hyperthermia after discontinuance of Levodopa and bromocriptine therapy: Impaired dopamine receptors a possible cause. Neurology.

[9] Ueda M, Hamamoto M, Nagayama H, Okubo S, Amemiya S, Katayama Y (2001). Biochemical alterations during medication withdrawal in Parkinson's disease with and without neuroleptic malignant-like syndrome. J Neurol Neurosurg Psychiatry.

[10] Ueda M, Hamamoto M, Nagayama H, Otsubo K, Nito C, Miyazaki T (1999). Susceptibility to neuroleptic malignant syndrome in Parkinson's disease. Neurology.

[11] Sato Y, Asoh T, Metoki N, Satoh K (2003). Efficacy of methylprednisolone pulse therapy on neuroleptic malignant syndrome in Parkinson's disease. J Neurol Neurosurg Psychiatry.

